# LPS induces limited activation of hypoxia-inducible factor-1α in macrophages

**DOI:** 10.1016/j.jbc.2025.110932

**Published:** 2025-11-11

**Authors:** Takayuki Isagawa, Masaki Suimye Morioka, Hiroaki Semba, Daigo Sawaki, Tatsuyuki Sato, Masaki Wake, Hiroki Sugimoto, Shigeru Sato, Kazutoshi Ono, Chuluun-Erdene Ariunbold, Thuc Toan Pham, Ryohei Tanaka, Toshinaru Kawakami, Masamichi Ito, Shun Minatsuki, Yasutomi Higashikuni, Hidemasa Bono, Hiroshi Harada, Masataka Asagiri, Ichiro Manabe, Christian Stockmann, Takahide Kohro, Takahiro Kuchimaru, Norihiko Takeda

**Affiliations:** 1 Data Science Center, Jichi Medical University, Shimotsuke, Japan; 2 Division of Bioconvergence, Center for Molecular Medicine, Jichi Medical University, Shimotsuke, Japan; 3 Preventive Medicine and Applied Genomics Unit, RIKEN Center for Integrative Medical Sciences, Yokohama, Kanagawa, Japan; 4 Research Fellow of Japan Society for the Promotion of Science; 5 Division of Nephrology, Department of Internal Medicine, Jichi Medical University, Shimotsuke, Japan; 6 Department of Cardiovascular Medicine, Graduate School of Medicine, The University of Tokyo, Bunkyo-ku, Tokyo, Japan; 7 Division of Cardiovascular and Genetic Research, Center for Molecular Medicine, Jichi Medical University, Shimotsuke, Japan; 8 Graduate School of Integrated Sciences for Life, Hiroshima University, Higashi-Hiroshima, Hiroshima, Japan; 9 Laboratory of Cancer Biology, Graduate School of Biostudies, Kyoto University, Kyoto, Japan; 10 Department of Pharmacology, Graduate School of Medicine and RICeD, Yamaguchi University, Yamaguchi, Japan; 11 Department of Systems Medicine, Chiba University Graduate School of Medicine, Chuo-ku, Chiba, Japan; 12 Institute of Anatomy, University of Zurich, Zurich, Switzerland

**Keywords:** hypoxia-inducible factor, lipopolysaccharide, hypoxia, macrophage, ChIP sequencing

## Abstract

Hypoxia-inducible factor-1α (HIF-1α) plays a crucial role in cellular and tissue adaptation to low oxygen conditions. Although inflammatory stimuli such as lipopolysaccharide (LPS) also increase HIF-1α levels under normoxia, its transcriptional activity and regulatory mechanisms in this context remain unclear. To address this, we performed chromatin immunoprecipitation sequencing and transcriptome analyses in murine macrophages stimulated with either LPS or hypoxia. Both stimuli stabilized HIF-1α protein but *via* distinct mechanisms: hypoxia acted post-translationally, whereas LPS increased *Hif-1α* mRNA expression. Genome-wide HIF-1α binding was observed under both conditions; however, only hypoxia induced broad transcriptional activation of target genes, whereas LPS upregulated a restricted set, mostly glycolytic genes. Motif enrichment analysis revealed that hypoxia, but not LPS, promoted cooperative transcription factor engagement, including HIF-1β, ETS, and bZIP family members. Hypoxia also increased H3K27 acetylation at HIF-1α target loci, consistent with a transcriptionally permissive chromatin state. In contrast, LPS led to reduced H3K27ac at noninduced loci, suggesting epigenetic repression. Mechanistically, HIF-1α exhibited a phosphorylation-dependent band shift under hypoxia but not LPS. Although both conditions showed comparable overall phosphorylation levels by Phos-tag analysis, only hypoxia triggered a conformational change, suggesting site-specific phosphorylation linked to transcriptional competence. These findings demonstrate that HIF-1α binding alone is insufficient for gene activation and that phosphorylation and chromatin context determine its transcriptional output in a stimulus-dependent manner.

Oxygen is essential for normal physiological functions in all eukaryotic cells. In response to hypoxia, cells activate a transcriptional program to maintain homeostasis, primarily through hypoxia-inducible factors HIF-1α and HIF-2α. These transcription factors promote glycolysis, angiogenesis, and cell survival to preserve oxygen homeostasis (1). Under normoxic conditions, prolyl hydroxylases (PHD1, PHD2, and PHD3; encoded by *Egln2*, *Egln1*, and *Egln3*, respectively) hydroxylate HIF-1α and HIF-2α, targeting them for degradation *via* the VHL ubiquitin E3 ligase complex (2, 3, 4). The interaction between HIF-1α and p300/CBP drives transcriptional activation (5), and post-translational modifications of HIF-1α fine-tune its transcriptional activity. Factor inhibiting HIF (FIH) hydroxylates a conserved asparagine residue of HIF-α and blocks interaction with the transcriptional coactivators p300/CBP (6, 7). In contrast, phosphorylation of HIF-α enhances the interaction with coactivators and accelerates its transcriptional activity (8).

In addition to its canonical role in hypoxic adaptation, HIF-1α plays a central role in innate immune responses, particularly in macrophages. During bacterial infection, macrophages are exposed to both inflammatory signals and metabolically stressed microenvironments characterized by limited oxygen availability, increased cellular respiration, and poor vascular perfusion. These conditions give rise to a localized inflammatory microenvironment in which macrophages must rapidly adapt their metabolic and functional states. HIF-1α is critical in this context, as it drives a shift toward glycolysis to support proinflammatory and antimicrobial responses (9, 10). Specifically, HIF-1α promotes the expression of inducible nitric oxide synthase, antimicrobial peptides such as cathelicidins, and enzymes involved in glycolysis, all of which contribute to bacterial clearance (9, 11). Moreover, HIF-1α enhances phagocytic capacity and lysosomal acidification, facilitating intracellular killing of pathogens. In murine models, HIF-1α-deficient macrophages show impaired bacterial clearance and increased susceptibility to pathogens, such as *Escherichia coli* and *Mycobacterium tuberculosis* (12). These findings underscore the critical role of HIF-1α in host defense during infection.

Interestingly, bacterial components such as lipopolysaccharide (LPS) can induce HIF-1α expression even under normoxic conditions, through pathways involving reactive oxygen species, succinate accumulation, and inhibition of PHD enzymes—a phenomenon termed “pseudohypoxia” (13, 14). This LPS-induced stabilization of HIF-1α supports a transcriptional program distinct from classical hypoxic responses and is partially mediated by NF-κB and mitogen-activated protein kinase signaling (15, 16). While this response is protective in acute infection, sustained or dysregulated HIF-1α activity can exacerbate inflammation, contributing to septic shock or chronic inflammatory pathologies.

Despite these advances, the transcriptional mechanisms by which HIF-1α operates in LPS-stimulated macrophages remain incompletely defined. Although HIF-1α binds hypoxia-responsive elements (HREs) containing the core sequence 5′-RCGTG-3′ (17, 18), this motif alone does not predict *in vivo* binding or functional output. Furthermore, whether LPS-stabilized HIF-1α is functionally equivalent to hypoxia-induced HIF-1α remains unclear. Clarifying these differences is essential for understanding how HIF-1α integrates inflammatory and metabolic signals in normoxic yet activated macrophages and whether post-translational regulation under inflammatory conditions diverges from the canonical hypoxic pathway. These unanswered questions underscore the need to investigate the molecular basis of HIF-1α activity under inflammatory stimulation, particularly in macrophages responding to LPS.

## Results

### Genome-wide identification of LPS- or hypoxia-induced HIF-1α-binding sites in thioglycollate-elicited peritoneal macrophages

**Figure 1:**
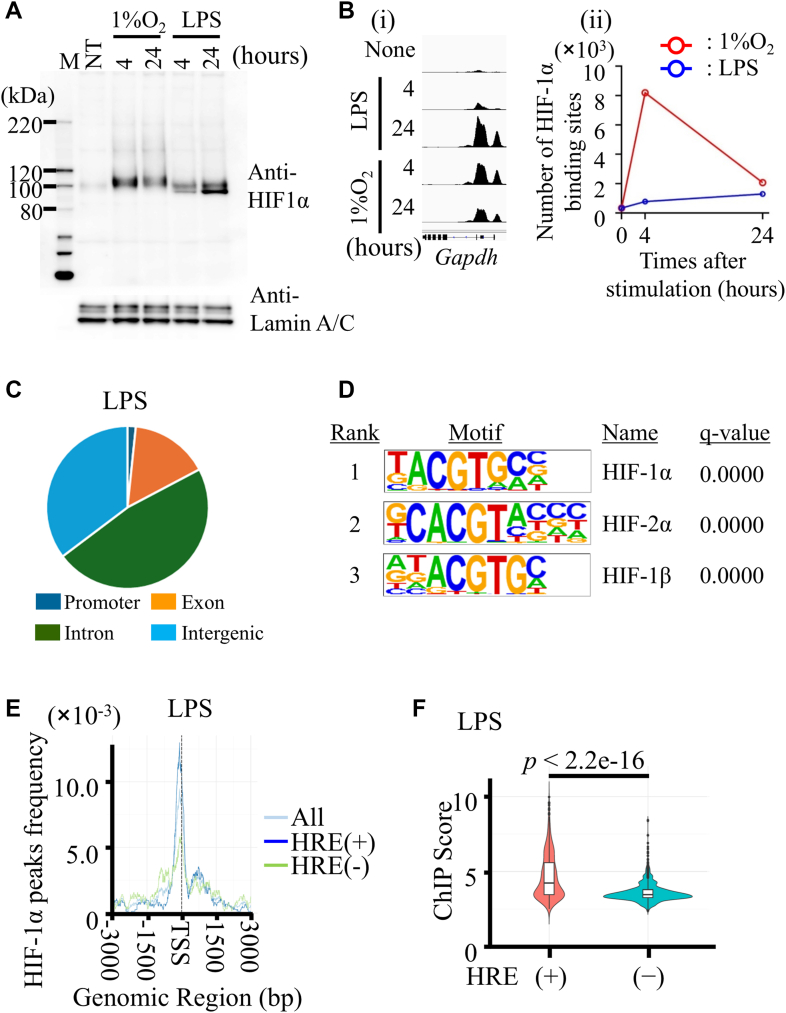
**ChIP-Seq analysis of HIF-1α in TEPMs under LPS stimulation and hypoxic conditions.***A*, Western blot analysis of HIF-1α protein levels in nuclear extracts at 0, 4, and 24 h following LPS stimulation or hypoxic exposure. *B*, (i) Integrative Genomics Viewer (IGV) visualization of HIF-1α binding at the *Gapdh* gene locus. (ii) Number of HIF-1α binding sites at 0, 4, and 24 h under LPS stimulation or hypoxic conditions. *C*, genomic distribution of HIF-1α binding sites identified by ChIP-Seq after LPS stimulation. *D*, transcription factors enriched in HIF-1α-bound motifs 24 h post-LPS stimulation. *De novo* motif analysis of the top 20 HIF-1α peaks identified the top three transcription factors, each matching the canonical HRE sequence. *E*, average HIF-1α peak profiles at transcription start sites. Binding signals within ±3 kb of the transcription start sites are shown for all peaks, HRE (+), and HRE (−) groups at 24 h post-LPS. *F*, comparison of HIF-1α peak intensities between HRE (+) and HRE (−) regions. Violin plots display log2-transformed ChIP-Seq peak scores calculated using FindPeaks. ChIP-Seq, chromatin immunoprecipitation sequencing; HIF-1α, hypoxia-inducible factor-1α; HRE, hypoxia-responsive element; LPS, lipopolysaccharide; TEPM, thioglycollate-elicited peritoneal macrophage.

To examine the subcellular localization and regulatory mechanisms of HIF-1α under hypoxic and LPS conditions, we analyzed HIF-1α protein expression in thioglycollate-elicited peritoneal macrophages (TEPMs) treated with 1 μg/ml LPS or exposed to 1% O_2_. Nuclear HIF-1α accumulation peaked at 24 h after LPS stimulation and at 4 h under hypoxia (Fig. 1*A*). In the cytoplasmic fraction, HIF-1α was detectable at 4 h after both treatments, with higher levels under hypoxia, and persisted under LPS at 24 h (Fig. S1).

To assess whether LPS or hypoxia differentially regulate HIF-1α gene expression and upstream signaling, we next measured *HIF-1α* mRNA levels and NF-κB activation. Quantitative RT–PCR (qRT–PCR) revealed that LPS stimulation markedly increased *HIF-1α* mRNA levels, whereas hypoxia did not elicit a detectable change (Fig. S2*A*). Consistently, Western blotting of nuclear fractions showed strong NF-κB (p65/RelA) activation in response to LPS but not hypoxia (Fig. S2*B*. These results suggest that LPS induces HIF-1α primarily through transcriptional upregulation mediated by NF-κB, whereas hypoxia acts *via* post-translational stabilization of the protein. To identify HIF-1α binding sites genomewide, we conducted chromatin immunoprecipitation sequencing (ChIP-Seq) analysis at 0, 4, and 24 h after LPS stimulation or hypoxia (Fig. 1*B*i). Consistent with the nuclear protein levels, the number of HIF-1α binding sites increased significantly following LPS stimulation (Fig. 1*B*, ii). We identified 1297 HIF-1α binding sites, with the highest number detected at 24 h post-LPS stimulation (Table 1). Under hypoxic conditions (1% O_2_), 8148 binding peaks were detected at 4 h poststimulation (Table 1). Among these hypoxia-induced peaks, 520 binding peaks overlapped with those identified following LPS stimulation (Fig. S3). This limited overlap highlights the distinct and stimulus-specific nature of HIF-1α binding. These data suggest that hypoxia and LPS stimulation engage largely nonoverlapping regulatory programs, resulting in unique HIF-1α cistromes under each condition. These binding sites exhibited nonrandom distribution, with enrichment in gene-associated regions: ∼8% in promoters, ∼10% in exons, and ∼82% in intronic or intergenic regions, a pattern similar to hypoxic conditions (Figs. 1*C* and S4*A*).

**Table 1:** Total HIF-1α binding sites under LPS and hypoxia count of peaks

Condition	LPS	1% O_2_
LPS	828	469
1% O_2_	469	7679

We next examined whether HIF-1α binding correlated with canonical HREs (5′-RCGTG-3′) using HOMER motif analysis. *De novo* analysis of the top 20 HIF-1α peaks at 24 h identified three major motifs (Fig. 1*D*), all containing the 5′-ACGTG-3′ sequence—consistent with the canonical HRE—while no clear motifs emerged at 0 or 4 h (Fig. S4*C* i and ii). HIF-1α frequently bound near transcription start sites, regardless of HRE presence (Fig. 1*E*). LPS-induced HIF-1α peaks containing HREs showed stronger ChIP-Seq signals than those lacking HREs (Fig. 1*F*). These results suggest that LPS promotes direct HIF-1α binding at gene promoters, whereas non-HRE sites may reflect indirect recruitment *via* other transcription factors or coactivators such as p300/CBP (19, 20, 21). Genes lacking HREs have not been previously identified as HIF-1α targets. Therefore, we focused subsequent analyses on genes with HRE-containing HIF-1α binding sites.

### HIF-1α binding does not correlate with gene expression under LPS stimulation

**Figure 2:**
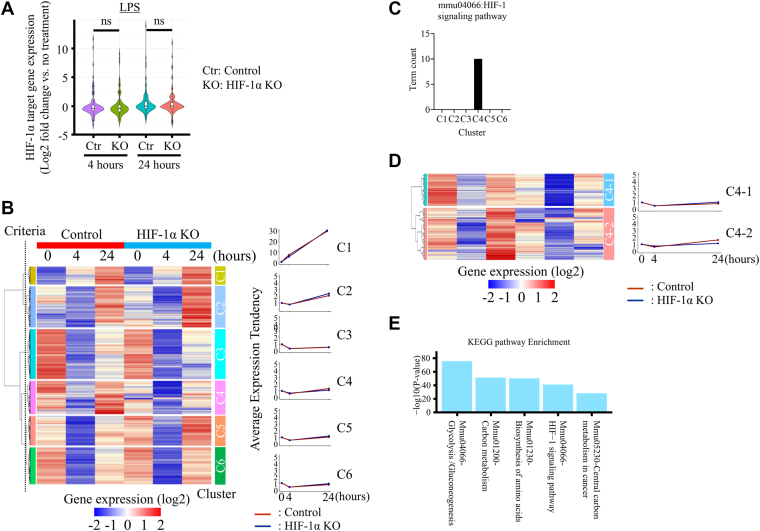

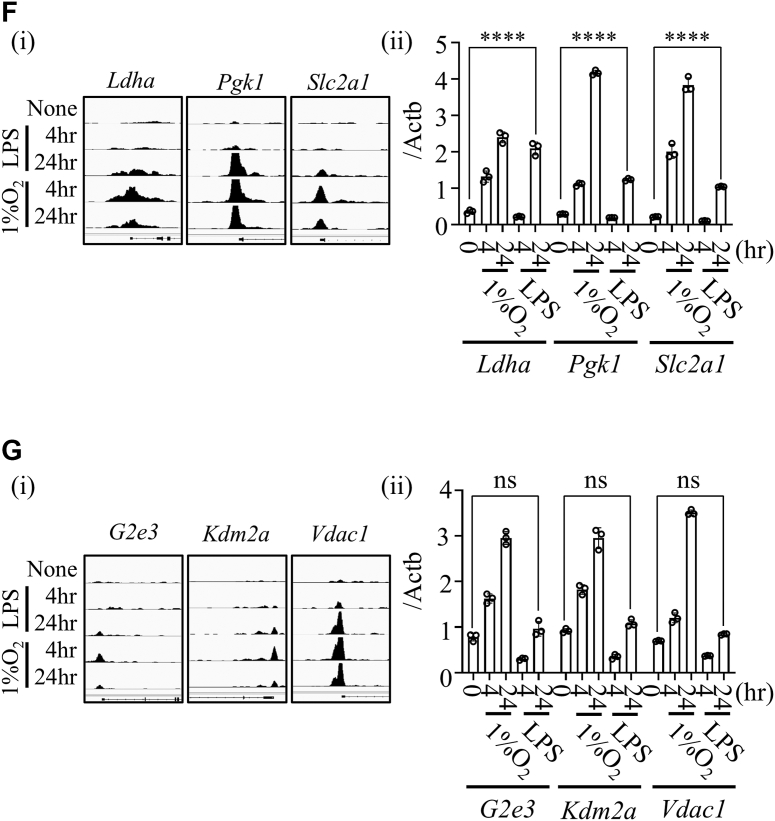
**HIF-1α binding under LPS does not induce target gene expression.***A*, comparison of LPS-induced HIF-1α target gene expression between WT and HIF-1α KO TEPMs. Violin plots show relative expression levels compared with untreated TEPMs. HIF-1α target genes under LPS stimulation were defined by HIF-1α binding at promoter HREs. *B*, hierarchical clustering of 903 genes with HIF-1α-bound promoters containing HREs, based on their expression profiles from 0 to 24 h after LPS stimulation. The *dashed line* indicates the clustering threshold (dendrogram height >0.6). *Line plots* show average expression in control (*red*) and HIF-1α KO (*blue*) TEPMs. *C*, KEGG pathway analysis using DAVID identified HIF-1 signaling as specifically enriched in cluster 4. *D*, subclustering of cluster 4 based on gene expression separated it into C4-1 and C4-2. *Line plots* show average expression for each subcluster in control and HIF-1α KO TEPMs. *E*, KEGG pathway analysis identified glycolysis and HIF-1 signaling as specifically enriched in C4-2. *F*, (i) IGV browser views of HIF-1α ChIP-Seq peaks at classical HIF-1α target genes (*Ldha*, *Pgk1*, and *Slc2a1*) under LPS and hypoxia. (ii) Quantitative expression analysis of classical HIF-1α target genes in TEPMs under LPS and hypoxic conditions. *G*, (i) IGV browser views at nonresponsive genes (*G2e3*, *Kdm2a*, and *Vdac1*). (ii) Quantitative expression analysis of nonresponsive genes in TEPMs under LPS and hypoxic conditions. *Asterisks* indicate statistical significance: ∗∗∗∗∗*p* < 0.0001; ns, not significant. *p* Values were determined by two-way ANOVA followed by Tukey's multiple comparisons test. Data are presented as the mean ± SD from technical triplicates. ChIP-Seq, chromatin immunoprecipitation sequencing; HIF-1α, hypoxia-inducible factor-1α; IGV, Integrative Genomics Viewer; KEGG, Kyoto Encyclopedia of Genes and Genomes; LPS, lipopolysaccharide; TEPM, thioglycollate-elicited peritoneal macrophage.

To assess whether HIF-1α binding correlates with nearby gene expressions under LPS stimulation, we performed transcriptome profiling of TEPMs isolated from hematopoietic/endothelial-specific *Hif-1α*-deficient mice (*Hif-1α* flox/flox; *Tie2-Cre* KO mice; HIF-1α KO) and Cre-negative littermate controls. We identified 903 genes with HIF-1α peaks at promoter HREs at 0, 4, and 24 h after LPS stimulation (Fig. S5*A*). Despite widespread HIF-1α binding, most target genes did not exhibit increased expression in response to LPS stimulation—a pattern not observed under hypoxic conditions (Figs. 2*A* and S5*A*). Hierarchical clustering grouped the 903 HIF-1α–bound genes into six distinct clusters (cluster 1 [C1]–C6) based on expression patterns (Fig. 2*B*, *left panel*). To further characterize the heterogeneity within C4, we performed additional hierarchical clustering, which revealed two distinct subgroups: C4-1 and C4-2. Genes in C4-2 showed clear HIF-1α–dependent induction, whereas those in C4-1 remained unresponsive despite HIF-1α binding. This classification was based on differences in gene expression between WT and HIF-1α KO macrophages. Furthermore, Kyoto Encyclopedia of Genes and Genomes (KEGG) pathway analysis revealed that the glycolysis and HIF-1 signaling pathways were specifically enriched in C4-2, but not in C4-1, supporting the biological significance of this subdivision (Fig. 2, *D* and *E*). These results indicate that under LPS stimulation, HIF-1α predominantly activates glycolytic genes, even though it binds broadly to many genomic sites. To functionally validate these transcriptomic findings, we assessed metabolic flux in TEPMs derived from Tie2-Cre-negative littermate controls and Tie2-Cre-positive HIF-1α KO mice using the Seahorse XF Analyzer after LPS stimulation (0, 4, and 24 h) (Fig. S6*A*). In control macrophages, oxygen consumption rate (OCR) transiently increased at 4 h but markedly decreased at 24 h, consistent with a shift toward glycolysis. In contrast, OCR remained significantly higher in HIF-1α KO macrophages at all time points and further increased at 24 h, showing the absence of the LPS-induced metabolic switch (Fig. S6*B*, *left*). Extracellular acidification rate was comparable between groups at 0 and 4 h but significantly decreased in HIF-1α KO macrophages at 24 h (Fig. S6*B*, right). These results confirm that HIF-1α is required for the LPS-induced glycolytic reprogramming of macrophages.

### HIF-1α stabilization alone is insufficient to induce target gene expression under LPS stimulation

To investigate why HIF-1α binding fails to activate transcription in most cases under LPS stimulation, we compared representative genes in each subcluster. We selected three HIF-1α–responsive genes (HRGs) (Fig. 2*F*, i) and ii)) from C4-2: *lactate dehydrogenase A* (*Ldha*), *phosphoglycerate kinase 1* (*Pgk1*), and *solute carrier family 2 member 1* (*Slc2a1*), and three HIF-1α–unresponsive genes (HURGs) (Fig. 2*G*), i) and ii)) from C4-1: *G2/M phase–specific E3 ubiquitin protein ligase* (*G2e3*), *lysine demethylase 2A* (*Kdm2a*), and *voltage-dependent anion channel 1* (*Vdac1*). HIF-1α bound the promoter regions of all six genes under both hypoxic and LPS conditions (Fig. 2, *F* i and *G* i).

**Figure 3:**
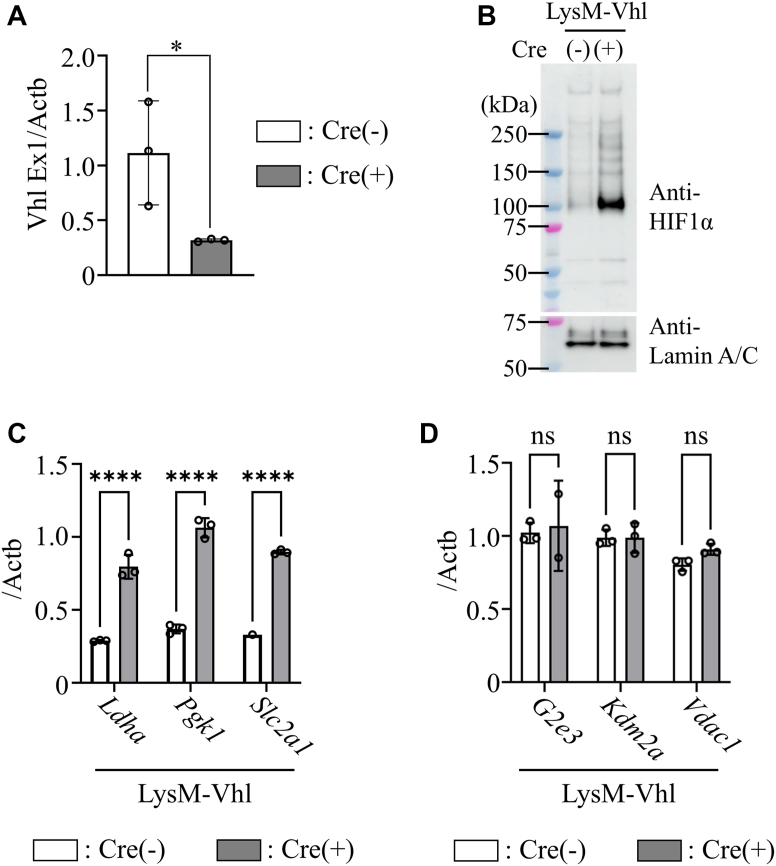
**Stabilization of HIF-1α protein by *Vhl* ablation does not induce target gene expression under LPS stimulation, except for glycolytic genes.***A*, deletion efficiency of *Vhl* mRNA in TEPMs (*Lysm-Cre* (+/−) *Vhl*^flox/flox^) was examined. *p* Values were determined by unpaired two-tailed *t* test. *B*, Western blot analysis of HIF-1α in nuclear extracts of TEPMs (*Lysm-Cre* (+/−) *Vhl*^flox/flox^). *C* and *D*, quantitative expression analysis of HIF-1α–responsive genes (HRGs) and HIF-1α–unresponsive genes (HURGs) in unstimulated TEPMs with or without *Vhl*. *Asterisks* indicate statistical significance: ∗*p* < 0.05; ∗∗∗∗∗*p* < 0.0001; ns, not significant. *p* Values were determined by two-way ANOVA followed by Šídák’s multiple comparisons test. Data are presented as the mean ± SD from technical triplicates. HIF-1α, hypoxia-inducible factor-1α; LPS, lipopolysaccharide; TEPM, thioglycollate-elicited peritoneal macrophage.

Since hematopoietic- and endothelial-specific *Vhl* KO is embryonically lethal, we used myeloid-specific *Vhl* KO mice to assess whether HIF-1α stabilization alone could induce HURG expression. *Vhl* deficiency stabilized HIF-1α protein, mimicking normoxic stabilization observed following LPS stimulation (Fig. 3, *A* and *B*). Although HIF-1α stabilization induced HRG expression in *Vhl*-deficient TEPMs even without LPS, HURG expression remained unchanged (Fig. 3, *C* and *D*).

**Figure 4:**
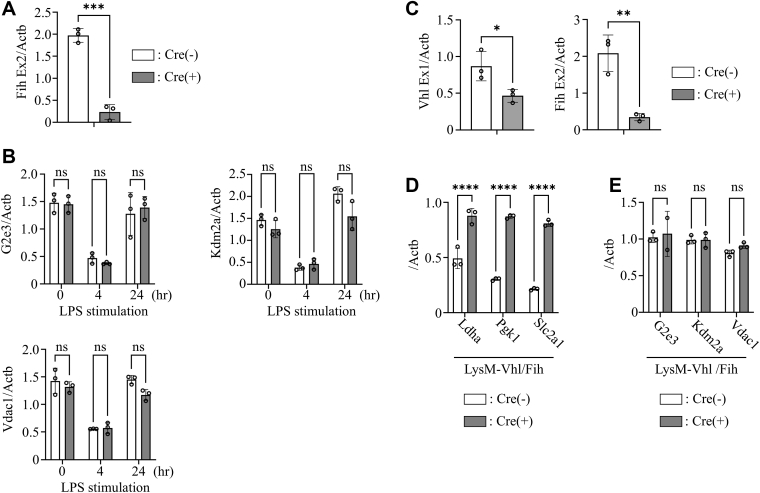
**Ablation of *Fih* in TEPMs does not induce target gene expression upon LPS stimulation.***A*, deletion efficiency of *Fih* mRNA in TEPMs (*Tie2-Cre* (+/−) *Fih*^flox/flox^) was examined. *p* Values were determined by unpaired two-tailed *t* test. *B*, Western blot analysis of Fih in cell lysates from TEPMs isolated from Tie2-Cre (+/−) *Fih*^flox/flox^ and LysM-Cre (+/−) *Vhl*^flox/flox^; *Fih*^flox/flox^ mice. *C*, quantitative expression analysis of HURGs in TEPMs with or without FIH under LPS stimulation. ns, not significant. *D*, deletion efficiency of *Vhl* and *Fih* mRNA in TEPMs (*Lysm-Cre* (+/−) *Vhl*^flox/flox^ and *Fih*^flox/flox^) was examined. *p* Values were determined by unpaired two-tailed *t* test. *D* and *E*, quantitative expression analysis of (*E*) HRGs and (*F*) HURGs in unstimulated TEPMs with or without *Vhl* and *Fih*. *Asterisks* indicate statistical significance: ∗*p* < 0.05; ∗∗*p* < 0.005; ∗∗∗∗∗*p* < 0.0001; ns, not significant. *p* Values were determined by two-way ANOVA followed by Sidak’s multiple comparisons test. Data are presented as mean ± SD from technical triplicates. FIH, factor inhibiting HIF; HRG, HIF-1α–responsive gene; HURG, HIF-1α–unresponsive gene; LPS, lipopolysaccharide; TEPM, thioglycollate-elicited peritoneal macrophage.

FIH negatively regulates HIF-1α transactivation by preventing coactivator recruitment. Because hematopoietic- and endothelial-specific *Fih* KO is viable, we examined whether FIH suppression affects HURG expression under LPS. LPS failed to induce HURG mRNA expression in *Fih*-deficient TEPMs (Fig. 4, *A* and *B*). Furthermore, myeloid-specific *Vhl*/*Fih* double KO in TEPMs (Fig. 4*D*) also failed to induce HURG expression (Fig. 4, *E* and *F*). These findings suggest that FIH plays no significant role in regulating the transcriptional activity of HIF-1α in macrophages.

### Motif enrichment analysis of HIF-1α–dependent genes under hypoxia and LPS stimulation

**Figure 5:**
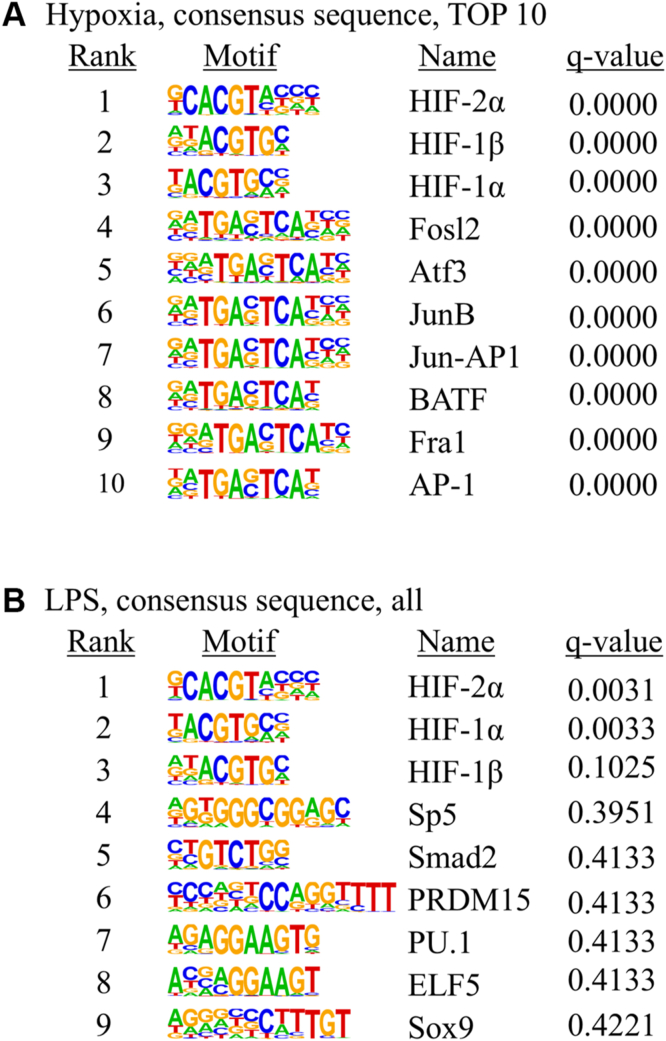
**Motif enrichment analysis of HIF-1α binding sites under hypoxia and LPS stimulation.***A*, top 10 enriched motifs under hypoxia. Motifs include canonical hypoxia-responsive elements (HREs; HIF-2α, HIF-1β, and HIF-1α) and cooperating transcription factors (ETS family, KLF, and AP-1). All the top 10 motifs were significantly enriched. *B*, consensus motifs identified under LPS stimulation (nine motifs). Only HIF-1α and HIF-2α motifs reached statistical significance, whereas other motifs—including inflammatory regulators (PU.1 and SMAD2) as well as SP5, PRDM15, ELF5, and SOX9—were detected but did not reach significance. HIF-1α–unresponsive gene; LPS, lipopolysaccharide.

Motif enrichment analysis was performed using HIF-1α–dependent genes upregulated under hypoxia or LPS stimulation. Under hypoxia, all the top 10 motifs were significantly enriched, with the top three corresponding to HIF-2α, HIF-1β, and HIF-1α. Additional enriched motifs included ETS family members, zinc finger proteins, and multiple bZIP/AP-1 family members (FOSL2, ATF3, JUNB, c-JUN, FRA1, FRA2, and BATF). This broad motif enrichment indicates that HIF-1α operates within a cooperative transcriptional network that integrates angiogenesis, metabolic adaptation, and stress/inflammatory responses (Fig. 5*A*).

In contrast, LPS stimulation yielded only nine significantly enriched motifs, primarily HIF-1α and HIF-2α. While other transcription factor motifs (including PU.1, SP5, and SMAD2) were detected, they did not reach statistical significance (Fig. 5*B*). These findings suggest that under LPS conditions, chromatin accessibility and coregulator recruitment are more restricted, thereby limiting HIF-1α transcriptional activity despite comparable binding events.

### Phosphorylation of HIF-1α differs between hypoxia and LPS stimulation

**Figure 6:**
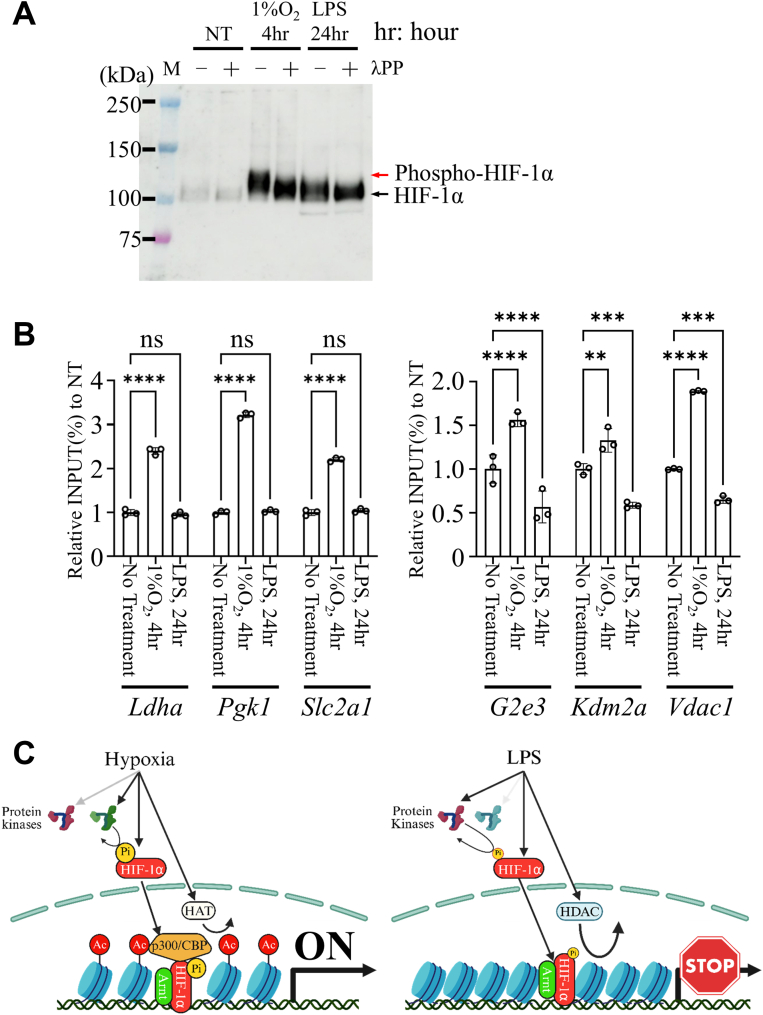
**Hypoxia and LPS differentially regulate H3K27 acetylation levels at HREs of HIF-1α target genes.***A*, Western blot analysis of HIF-1α protein in nuclear extracts of TEPMs under hypoxic conditions with or without λ-phosphatase (λPP) treatment. *B*, ChIP–qPCR analysis of H3K27ac at HREs within the promoter regions of HRGs and HURGs in TEPMs. Data are presented as mean ± SD. *Asterisks* indicate statistical significance: ∗∗*p* < 0.005; ∗∗∗*p* < 0.0005; ∗∗∗∗∗*p* < 0.0001; ns, not significant. *p* Values were determined by two-way ANOVA followed by Dunnett's multiple comparisons test. Data are presented as mean ± SD from technical triplicates. *C*, schematic illustration of stimulus-specific phosphorylation that differentially regulates HIF-1α activity under hypoxia and LPS stimulation. *Left panel (hypoxia),* hypoxia stabilizes HIF-1α and promotes its phosphorylation by a specific kinase at activation sites. This phosphorylation enables HIF-1α to interact with the transcriptional coactivator p300/CBP, thereby enhancing H3K27 acetylation at hypoxia-responsive elements (HREs) and activating transcription of hypoxia-responsive genes. *Right panel (LPS),* in contrast, LPS stimulation induces phosphorylation of HIF-1α by a distinct kinase at different residues, resulting in a transcriptionally inert form that fails to recruit p300/CBP or enhance histone acetylation. LPS stimulation also activates histone deacetylases (HDACs), reducing H3K27 acetylation at nonglycolytic HIF-1 target loci and suppressing their transcription. Ac, acetylation; ChIP, chromatin immunoprecipitation; HDAC, histone deacetylase; HIF-1α, hypoxia-inducible factor-1α; HURG, HIF-1α–unresponsive gene; LPS, lipopolysaccharide; Pi, phosphorylation; qPCR, quantitative PCR; TEPM, thioglycollate-elicited peritoneal macrophage.

HIF-1α induced by hypoxia migrated more slowly than LPS-induced HIF-1α, suggesting altered post-translational modification (Fig. 1*A*). To test whether this mobility shift reflects phosphorylation, we treated nuclear extracts with λ-phosphatase. λ-phosphatase treatment increased the mobility of hypoxia-induced HIF-1α but did not affect the LPS-induced form (Fig. 6*A*), indicating that hypoxia and LPS elicit distinct phosphorylation states.

To further examine HIF-1α phosphorylation, we performed Phos-tag Western blotting using nuclear extracts from unstimulated, hypoxia-treated (4 h), and LPS-treated (24 h) macrophages. Phosphorylated HIF-1α was detected under both conditions, with comparable band shifts, indicating similar overall phosphorylation levels. These results suggest that the functional differences arise not from the extent of phosphorylation but from site-specific modifications that differ between hypoxia and LPS (Fig. S7).

To assess the chromatin context of stimulus-specific HIF-1α activity, we performed ChIP–qPCR analysis for H3K27ac, a well-established marker of transcriptionally active promoters and enhancers. H3K27ac is dynamically regulated by environmental stimuli such as hypoxia and reflects p300/CBP-mediated histone acetylation, which is directly influenced by HIF-1α. Based on our hypothesis that HIF-1α phosphorylation promotes coactivator recruitment and chromatin activation, we selected H3K27ac as a representative epigenetic readout.

We then examined H3K27ac levels at HIF-1α target loci. Under hypoxic conditions, H3K27ac levels increased at HREs in both HRGs and HURGs, whereas LPS had no detectable effect (Fig. 6*B*). These results support the idea that hypoxia-induced phosphorylation of HIF-1α promotes coactivator recruitment, leading to increased histone acetylation and transcriptional activation.

## Discussion

This study investigated how HIF-1α regulates gene expression in TEPMs under inflammatory conditions. To identify genes both bound and transcriptionally activated by HIF-1α, we combined ChIP-Seq with transcriptomic profiling under LPS stimulation and hypoxia. HIF-1α binding increased genomewide, peaking at 24 h post-LPS and 4 h under hypoxia, in line with protein accumulation. Although both stimuli stabilized HIF-1α, only hypoxia broadly induced target gene expression, whereas LPS primarily activated glycolytic genes. These findings indicate that distinct signaling contexts shape stimulus-specific HIF-1α transcriptional outputs.

Notably, most LPS-induced HIF-1α binding events failed to activate transcription, despite the established role of HIF-1α in inflammation. Since inflamed tissues often become hypoxic because of vascular collapse, such transcriptionally silent binding may function as a priming mechanism for subsequent hypoxic adaptation. Consistent with these findings, our motif enrichment analysis revealed striking differences between hypoxia and LPS. Under hypoxia, HIF-1α–dependent promoters showed significant enrichment not only for HIF motifs (HIF-2α, HIF-1β, and HIF-1α) but also for ETS factors, zinc finger proteins, and bZIP/AP-1 family members, such as ATF3, JUNB, and BATF—reflecting a cooperative transcriptional network supported by an open chromatin state and efficient coactivator recruitment. In contrast, LPS stimulation yielded a limited motif profile, with significant enrichment largely restricted to HIF motifs. Although motifs for PU.1, SP5, and SMAD2 were detected, they did not reach statistical significance. These results indicate that HIF-1α likely achieves full transcriptional activation under hypoxia through cooperative interactions with other transcriptional partners such as ETS family members, AP-1, and zinc finger proteins, which are known coregulators of HIF-mediated gene expression (22, 23, 24, 25). In contrast, the absence of these coactivating partners under LPS stimulation may result in only partial activation of HIF-1α target genes. This differential cofactor engagement provides a mechanistic explanation for the distinct transcriptional outputs observed between hypoxia and LPS.

Consistent with these findings, our additional analyses revealed that LPS and hypoxia activate HIF-1α through distinct mechanisms. Whereas hypoxia primarily induces post-translational stabilization and nuclear accumulation of HIF-1α, LPS stimulation promotes Hif1a transcription *via* NF-κB activation, leading to cytoplasmic accumulation of HIF-1α with limited nuclear translocation. This mechanistic divergence likely explains the partial activation of HIF-1α–dependent transcription under LPS despite comparable protein stabilization. Interestingly, recent studies have suggested that cytoplasmic HIF-1α may regulate glycolytic flux independently of its transcriptional role (26). Our observation of persistent cytoplasmic HIF-1α under LPS stimulation raises the possibility that noncanonical, cytoplasmic functions of HIF-1α may contribute to metabolic adaptation under inflammatory conditions—a hypothesis that warrants further investigation.

Our data identify phosphorylation as a key determinant of HIF-1α transcriptional activity. Hypoxia, but not LPS, promoted a phosphorylation-associated mobility shift of HIF-1α on SDS-PAGE, accompanied by its interaction with the coactivator p300/CBP. This interaction likely reflects phosphorylation-dependent displacement of FIH, which otherwise blocks coactivator binding (18, 27, 28). Although LPS stabilized HIF-1α, it failed to provide the structural modification required for transcriptional activation. FIH deficiency alone was insufficient to restore gene induction at nonresponsive HIF-1α–bound loci, indicating that phosphorylation—not merely loss of FIH—is essential for full transcriptional competence. Although FIH regulates HIF in other cell types, its impact appears limited in macrophages, likely reflecting relatively low *Fih* expression in these cells (BioGPS). In contrast to skeletal muscle or T cells, where *Fih* deletion enhances HIF-driven metabolic alteration and immune responses (29, 30).

To further explore HIF-1α phosphorylation, we performed Phos-tag Western blotting and found that HIF-1α was phosphorylated under both hypoxia and LPS. However, only hypoxia induced a detectable band shift on SDS-PAGE, suggesting conformational changes driven by phosphorylation at distinct sites. A phosphospecific antibody against Ser641/Ser643 yielded negative results, implying that other residues are involved. Although phosphoproteomic analysis would be the most definitive method to identify these sites, it remains technically challenging in our system. Thus, the specific residues and kinases responsible for this modification remain to be determined, representing a key direction for future investigation. In line with these findings, pharmacological inhibition of kinases previously reported to phosphorylate HIF-1α—including PKA, ERK1/2, and p38 mitogen-activated protein kinase—neither did it affect the hypoxia-induced mobility shift observed on SDS-PAGE nor did it alter overall phosphorylation levels as assessed by Phos-tag analysis (data not shown). These results further support the notion that noncanonical or as-yet-unidentified kinases are responsible for the activating phosphorylation of HIF-1α under hypoxia. Elucidating these upstream regulators will be crucial to fully understand how environmental cues fine-tune HIF-1α function in macrophages.

Interestingly, several glycolytic genes—such as *Ldha*, *Slc2a1*, and *Pgk1*—were robustly induced by LPS despite the absence of detectable HIF-1α phosphorylation. This suggests that phosphorylation is not universally required for HIF-1α–mediated transcription. Previous studies have reported that certain glycolytic targets possess intrinsically high responsiveness to HIF-1α and can be activated even when coactivator recruitment *via* p300/CBP is impaired. Consistent with this notion, our ChIP–qPCR analysis showed that H3K27ac levels at glycolytic loci remained stable under LPS stimulation, suggesting a permissive chromatin state that allows transcriptional activation despite limited coactivator engagement. In contrast, nonresponsive, hypoxia-inducible genes exhibited reduced H3K27ac under LPS stimulation, indicating active chromatin repression. These observations suggest that the limited transcriptional response to LPS is not only solely because of the absence of HIF-1α phosphorylation but also reflects an epigenetic environment that restricts gene activation. Notably, LPS has been shown to increase histone deacetylase 2 activity, which promotes chromatin compaction and transcriptional silencing (31, 32). The decrease in H3K27ac at nonresponsive loci raises the possibility that histone deacetylase 2 antagonizes histone acetyltransferase activity at these sites. Thus, HIF-1α–independent chromatin-modifying pathways may contribute to the selective repression of target genes under inflammatory conditions.

Together, our findings define phosphorylation as a molecular switch that converts HIF-1α from a stabilized but transcriptionally inactive form under LPS into a fully competent regulator under hypoxia. Stabilization alone—*via* PHD inhibition, VHL deletion, or FIH loss—cannot substitute for this regulatory step. Phosphorylation thus serves as a critical control point that integrates environmental cues into transcriptional output. By delineating how stimulus-specific signaling and chromatin context converge to regulate HIF-1α function, this study lays a foundation for future research aimed at identifying upstream kinases and epigenetic modifiers that fine-tune macrophage responses in inflammation and hypoxia.

## Experimental procedures

### Mice

All animal experiments followed protocols approved by the ethics committee for animal experiments at Jichi Medical School and the University of Tokyo. WT C57BL/6J mice were purchased from CLEA Japan. B6.129-*Hif1α*^tm3Rsjo^/J (*Hif-1α flox/flox*, JAX, stock no.: #007561) mice, B6.129S4(C)-*Vhl*^tm1Jae^/J (*Vhl flox/flox*, JAX, stock no.: 012933) mice, B6.Cg-Tg(*Tek-cre*)1Ywa/J (*Tie2*-*Cre*, JAX, stock no.: 008863) mice and B6.129P2-*Lyz2*^tm1(cre)Ifo^/J (*LysM-Cre*, JAX, stock no.: 004781) mice were purchased from The Jackson Laboratory and *Fih flox/flox* mice (*F. flox/flox*), which contain loxP sites flanking exon 2 of *Fih*, from Professor Randall S. Johnson (University of Cambridge, United Kingdom).

We bred homozygous *Fih flox/flox* or *Vhl flox/flox* mice to generate respective floxed strains. To generate hematopoietic- and endothelial-specific or myeloid-specific deletions, we crossed these mice with *Tie2-Cre* or *LysM-Cre* mice. This breeding strategy produced conditional KOs: HIF-1α KO, VHL KO, FIH KO, and VHL–FIH double KO. We isolated total RNA from TEPMs and assessed gene deletion efficiency using qPCR with primers spanning the target region and an undeleted control gene for normalization (Table 2). Cre-negative homozygous littermates served as controls. We used female mice aged 7 to 12 weeks for all experiments.

**Table 2:** List of primers used for qRT–PCR and ChIP–qPCR primers

qRT–PCR primer name	F/R	5′-sequence-3′
*Ldha*	F	TGGCAGACTTGGCTGACAG
	R	ACCTTCACAACATCCGAGATTC
*Pgk1*	F	CTGTGGTACTGAGAGCAGCAAGA
	R	CAGGACCATTCCAAACAATCTG
*Slc2a1*	F	GGACCCTGCACCTCATTG
	R	GCCACGATGCTCAGATAGG
*G2e3*	F	CGCGTATCAAGAGCTGCTG
	R	CTGTTAGGTTCGTTGTAGTCTC
*Kdm2a*	F	GTATCACATCCACCAAGGTG
	R	CTGTTTCCCTGATAGCAGC
*Vdac1*	F	TCAGGTCGACCCTGATGC
	R	CTGACAACGTCAGTTTGATACCT


ChIP primer name	F/R	5′-sequence-3′
*Ldha*	F	TGAGGCTGAGGAGCATGTC
	R	CACGATGTCCCTGCAAGAGT
*Pgk1*	F	CAACAAGCTGACTTTGGACAAG
	R	CAGTACGGAATTACCTCATCAC
*Slc2a1*	F	ATTTCTAAGGCCCTGGGTCC
	R	CCTGCCTGATGCGTGTCA
*G2e3*	F	GACAATAGGGAAGTGTGATG
	R	GCTTGCCCATGCTAATGAG
*Kdm2a*	F	GAACTGGAAAGGTGCTCTG
	R	ATCCTCCTGTGTGAGGGA
*Vdac1*	F	CACGCTCAACAGGGCAGGA
	R	GCAGTGTAGAGAGGGCGGA

F, forward; R, reverse.

### Cell culture

We isolated TEPMs from the peritoneal cavity 4 days after injecting mice intraperitoneally with a 3% thioglycollate solution (Fluka, Sigma–Aldrich). We cultured TEPMs in RPMI1640 (WAKO) supplemented with 1% (v/v) penicillin–streptomycin (WAKO) and 10% fetal bovine serum (BIOWEST) at 37 °C in a humidified CO_2_ incubator. We stimulated cells with LPS (Sigma; 1 μg/ml) in a standard incubator. For hypoxic exposure (1% O_2_), we used a personal CO_2_ multigas incubator (APM-30D; ASTEC) calibrated to maintain 1% O_2_, 5% CO_2_, and 94% N_2_ under a continuous nitrogen flow. Normoxic conditions (21% O_2_, 5% CO_2_) were maintained in a standard tissue culture incubator.

### Western blotting

We prepared cytoplasmic and nuclear extracts from cells cultured under normoxia, hypoxia, or LPS stimulation using the NE-PER Nuclear and Cytoplasmic Extraction Kit (Thermo Scientific). We solubilized fractions in sampling buffer (20 mM Tris, pH 7.4, 150 mM NaCl, 1 mM EDTA, 1 mM EGTA, 1% Triton X-100, protease inhibitor cocktail, and phosphatase inhibitor cocktail). We separated 10 μg of nuclear protein on 4% to 8% Tris–acetate gels (Thermo Scientific) and transferred proteins onto Immobilon-P polyvinylidene fluoride membranes (Merck Millipore). After blocking with 5% nonfat dry milk in Tris-buffered saline with 0.05% Tween-20, we incubated membranes with primary antibodies, followed by horseradish peroxidase–conjugated secondary antibodies. We detected protein signals using Amersham ECL Select (Cytiva) or ECL Prime (Cytiva). The antibodies used were anti-HIF-1α (NB100-449; Novus Biologicals), anti-Lamin A/C (#2032; Cell Signaling Technology), and horseradish peroxidase–conjugated goat anti-rabbit immunoglobulin G (sc-2004; Santa Cruz Biotechnology). To assess the phosphorylation status of HIF-1α, we treated nuclear extracts from TEPMs—prepared after hypoxia or LPS stimulation—with λ-phosphatase (sc-200312; Santa Cruz Biotechnology) at 30 °C for 120 min.

### Quantitative RT–PCR

We extracted total RNA using the RNeasy Mini Kit (Qiagen) and synthesized complementary DNA using the ReverTra Ace qPCR RT Master Mix (TOYOBO). We performed qPCR with the THUNDERBIRD SYBR qPCR MIX kit (TOYOBO) and confirmed single PCR product amplification *via* melting curve analysis. We used the standard curve method for quantification, preparing 5-, 25-, 125-, and 625-fold dilutions. We generated a standard curve from Ct values and calculated gene expression accordingly. We normalized all gene expression levels to Actb (β-actin). Primer sequences are listed in Table 2.

### ChIP assay

We performed ChIP as previously described (27, 28). Briefly, cells were crosslinked for 10 min using 1% paraformaldehyde and then subjected to fragmentation using Sonifier (Branson). We immunoprecipitated samples using the HIF-1α antibody (NB100-134; Novus Biologicals) and the acetyl-histone H3 (Lys27) antibody (AB_2561016; Active Motif). We then quantified ChIP samples by qPCR with specific primer pairs. Table 2 lists all mouse primer sequences for PCR.

### mRNA expression profiling

We collected TEPMs at 0, 4, and 24 h after LPS (1 μg/ml) or hypoxic (1% O_2_) exposure for transcriptome analysis (RNA-Seq). We prepared single-end RNA-Seq libraries using the TruSeq RNA Sample Prep Kit (Illumina). Sequencing runs were performed on an Illumina Genome Analyzer IIx (Illumina).

### Next-generation sequencing data processing

Raw FASTQ files were assessed with FastQC (v0.11.9), and sequencing adapters were removed with Cutadapt (v2.8). Adapter-trimmed reads were mapped to the mouse reference genome (GRCm38) with STAR (v2.7.3a), using the GENCODE vM25 gene annotation. Gene expression values were obtained with featureCounts (v2.0.0) and converted to log_2_-transformed transcripts per million with the edgeR package in R (v4.2.0).

For ChIP-Seq analysis, reads were aligned to GRCm38 with BWA (version 0.7.17); PCR duplicates were removed with Picard tools, and alignments with mapping quality <20 were discarded with SAMtools (version 1.10). All downstream steps were performed with the HOMER suite (version 4.11.1). HIF-1α peaks were called with findPeaks (false discovery rate = 0.0001) using reads from the HIF-1α-KO sample as control. We considered peaks with scores >20 to be definitive HIF-1α peaks and used them exclusively for peak-number quantification. Finally, peak locations were annotated and integrated with gene-expression data using mergePeaks.pl and annotatePeaks.pl together with the GENCODE vM25 annotation.

### Permutation test for cluster separation

The statistical significance of subdividing C4 into two subgroups (C4-1 and C4-2) was evaluated using a permutation test based on the difference between the mean between-cluster and within-cluster distances. For each of 999 permutations, the cluster labels were randomly reassigned while preserving the original subgroup sizes, and the statistic was recalculated. The *p* value was defined as the proportion of permutations in which the permuted statistic exceeded or equaled the observed value.

### *De novo* motif analysis and HRE motif search

To evaluate DNA motif enrichment in LPS-induced HIF-1α peaks, we used findMotifsGenome.pl with default parameters. We identified canonical HREs within LPS-induced peaks using annotatePeaks.pl with a custom RCGTG motif (17).

To identify potential coregulatory transcription factors associated with HIF-1α–binding sites, motif enrichment analysis was performed using HOMER (findMotifs.pl). Genes showing more than twofold upregulation in HIF-1α WT compared with KO macrophages under LPS or hypoxia were defined as HIF-1α–dependent genes. The nearest HIF-1α–binding peaks were assigned to these genes, and sequences within the peaks were analyzed.

For comparison of stimulus-specific programs, *cis*-regulatory regions of genes upregulated more than twofold in LPS *versus* hypoxia (n = 17) or in hypoxia *versus* LPS (n = 315), and also upregulated in WT relative to KO, were analyzed in the same way. Background sequences were generated automatically by HOMER from regions matched for GC content and length. Motif enrichment significance was evaluated using the cumulative binomial distribution implemented in HOMER.

### ChIP-Seq peak calling and data integration

We processed data using the HOMER suite (version 4.11.1). We identified HIF-1α peaks using findPeaks with a false discovery rate of 0.0001, using HIF-1α KO reads as a control. HIF-1α peaks were merged with mergePeaks (options: -d = given), and integrated gene expression with HRE annotations using annotatePeaks.pl.

### HIF-1a-bound HRE gene expression table and hierarchical clustering

After removing rows with NA values in the gene expression data, we extracted genes with HIF-1α peaks that overlapped an HRE motif within the region spanning −1500 to +500 bp from the transcription start site. To exclude genes with no variation in expression, genes with a standard deviation of zero across samples were removed. To capture gene expression changes induced by LPS between 4 and 24 h, we extracted genes whose expression at 24 h was more than 1.2-fold higher than at 4 h. Finally, hierarchical clustering was performed using the pheatmap function in R (distance = "euclidean" and method = "ward.D"), with an initial cut height of h = 50 and a second-level cut into k = 2 clusters.

### Gene Ontology and KEGG pathway enrichment analysis

We input HIF-1α-bound HRE promoter gene lists into the DAVID Bioinformatics Suite for Gene Ontology biological process and KEGG pathway enrichment analysis (31). We adjusted *p* values using the Benjamini–Hochberg method.

### Metabolic flux analysis

OCR and extracellular acidification rate were measured using the Seahorse XF24 Extracellular Flux Analyzer (Agilent Technologies) to assess mitochondrial respiration and glycolytic activity in TEPMs. Details of the experimental procedures and statistical analyses are provided in the supporting information.

### Statistical analysis

We present results as means ± SD. We used unpaired two-tailed *t* test and two-way ANOVA with Tukey’s, Šídák’s, or Dunnett’s multiple comparisons tests in GraphPad Prism 9 (GraphPad Prism Software, Inc). We considered *p* < 0.05 statistically significant.

## Data availability

The RNA-Seq and ChIP-Seq datasets for peritoneal macrophages have been deposited in the DNA Data Bank of Japan BioProject under accession number DRA008230 and in the National Center for Biotechnology Information Short Read Archive under accession number PRJNA1244111.

## Supporting information

This article contains supporting information.

## Conflict of interest

The authors declare that they have no conflicts of interest with the contents of this article.
