# Supplementary material for: LPS induces limited activation of hypoxia-inducible factor-1α in macrophages
Source: J Biol Chem. 2025 Nov 11;301(12):110932. doi: 10.1016/j.jbc.2025.110932 (PMC12720325; doi:10.1016/j.jbc.2025.110932)
Supplement: Supporting Figures [file mmc1.docx]

Supporting Information for

LPS Induces Limited Activation of Hypoxia-Inducible Factor-1α (HIF-1α) in Macrophages

Takayuki Isagawa*, Masaki Suimye Morioka, Hiroaki Semba, Daigo Sawaki, Tatsuyuki Sato, Masaki Wake, Hiroki Sugimoto, Shigeru Sato, Kazutoshi Ono, Chuluun-Erdene Ariunbold, Thuc Toan Pham, Ryohei Tanaka, Toshinaru Kawakami, Masamichi Ito, Shun Minatsuki, Yasutomi Higashikuni, Hidemasa Bono, Hiroshi Harada, Masataka Asagiri, Ichiro Manabe, Christian Stockmann, Takahide Kohro, Takahiro Kuchimaru and Norihiko Takeda*

*Correspondence: Takayuki Isagawa, i-takayuki13@jichi.ac.jp and Norihiko Takeda, ntakeda-tky@g.ecc.u-tokyo.ac.jp

This file includes:

Supplementary Methods.

Supplementary Figures S1 to S7 and the accompanying figure legends.

**Supplementary Methods**

**Metabolic flux analysis (Seahorse XF24)**

Oxygen consumption rate (OCR) and extracellular acidification rate (ECAR) were measured using the Seahorse XF24 Extracellular Flux Analyzer (Agilent Technologies, USA). Thioglycollate-elicited peritoneal macrophages (TEPMs) from control and Tie2-Cre–mediated *Hif-1α*–deficient (HIF-1α KO) mice were seeded at 2 × 10^5^ cells per well in XF24 cell culture plates in RPMI 1640 supplemented with 10% FBS. Cells were stimulated with 1 μg/mL LPS for 0, 4, or 24 hours, and measurements were performed using Seahorse XF Assay Medium according to the manufacturer’s instructions.

OCR and ECAR data were normalized to cell number and analyzed using Wave Desktop Software (Agilent). Basal OCR and ECAR values were compared between groups using two-way ANOVA followed by Šídák’s multiple comparisons test. Data are shown as mean ± SD; p < 0.05 was considered statistically significant.

**Phos-tag SDS–PAGE and Western blot analysis**
Phosphorylation of HIF-1α was analyzed using Phos-tag SDS–PAGE as well as conventional SDS–PAGE. Thioglycollate-elicited peritoneal macrophages (TEPMs) were stimulated with 1 μg/mL LPS for 24 hours or exposed to 1% O₂ for 4 hours, and nuclear extracts were prepared using NE-PER Nuclear and Cytoplasmic Extraction Reagents (Thermo Fisher Scientific).

Proteins (10 μg) were separated on 7.5% SuperSep™ Phos-tag gels (FUJIFILM Wako Pure Chemical Corporation) according to the manufacturer’s instructions. For comparison, equal amounts of protein were also analyzed by standard SDS–PAGE. Following electrophoresis, gels were treated with EDTA-containing transfer buffer and transferred to PVDF membranes (Immobilon-P; Millipore).

Membrane blocking, antibody incubation, and signal detection were performed as described in the main Methods section (“Western blot analysis”).

Phosphorylation-dependent mobility shifts of HIF-1α were compared between standard and Phos-tag gels to evaluate differences between hypoxia- and LPS-induced modifications.

**Supplementary Figures S1 to S7 and the accompanying figure legends.**


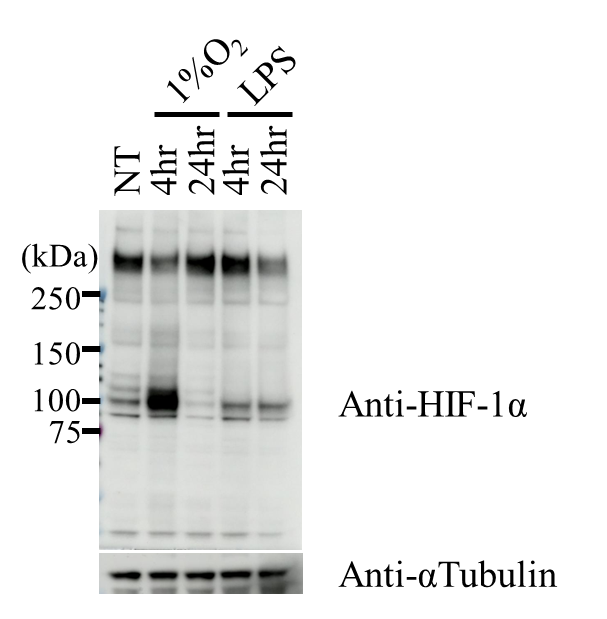
**Figure S1. Western blot analysis of HIF-1α protein levels in cytoplasmic extracts following LPS stimulation or hypoxic exposure.**

Cytoplasmic extracts from thioglycollate-elicited peritoneal macrophages (TEPMs) were collected at 0, 4, and 24 hours after treatment with 1 μg/mL LPS or exposure to 1% O₂. Protein levels of HIF-1α were analyzed by Western blotting using an anti–HIF-1α antibody (NB100-449, Novus Biologicals) and an anti–αTubulin antibody (66031-1-Ig, Proteintech). Under hypoxic conditions, cytoplasmic HIF-1α accumulation was observed at 4 hours but diminished by 24 hours. In contrast, LPS stimulation led to sustained cytoplasmic accumulation of HIF-1α at both 4 and 24 hours, with comparable intensities at the two time points. α-Tubulin was used as a loading control.

**
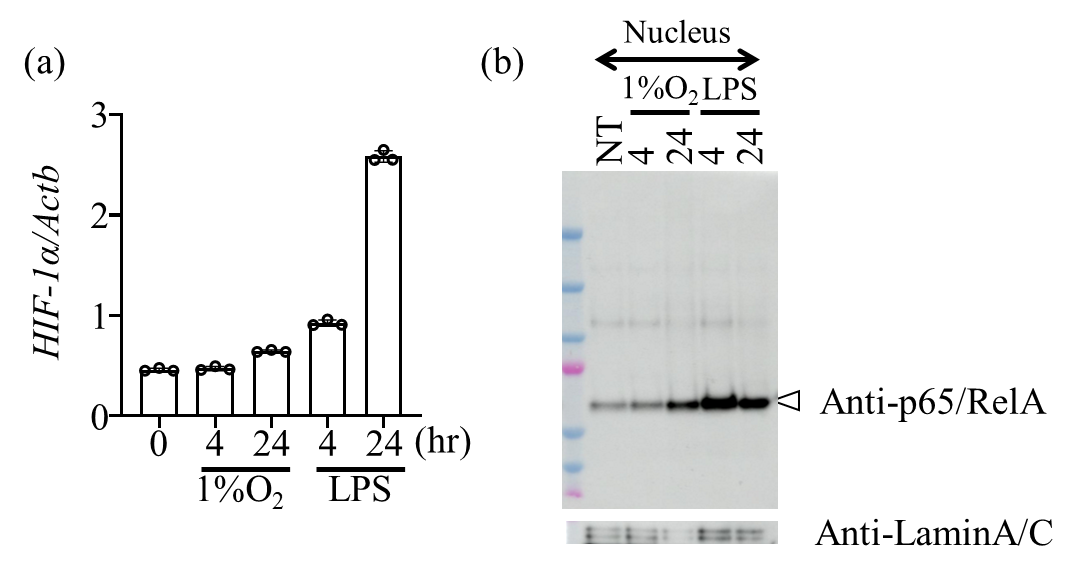
Figure S2. Distinct regulatory mechanisms of HIF-1α induction under LPS and hypoxia.**

(a) Quantitative RT–PCR analysis of Hif-1α mRNA expression in thioglycollate-elicited peritoneal macrophages (TEPMs) treated with 1 μg/mL LPS or cultured under 1% O_2_. LPS stimulation led to a gradual increase in *Hif-1α* transcript levels, which became prominent at 24 hours, whereas hypoxia did not elicit a detectable change. These results indicate that LPS and hypoxia regulate HIF-1α through distinct mechanisms—transcriptional versus post-translational regulation. (b) Western blot analysis of nuclear extracts from thioglycollate-elicited peritoneal macrophages (TEPMs) treated with 1 μg/mL LPS or exposed to 1% O₂ for the indicated time points (0, 4, and 24 hours). Nuclear translocation of the NF-κB p65/RelA subunit was examined as a marker of NF-κB activation using an anti–NF-κB p65 antibody (sc-372, Santa Cruz Biotechnology). Lamin A/C was detected using an anti–Lamin A/C antibody (#2032, Cell Signaling Technology) as a nuclear loading control. Under LPS stimulation, nuclear accumulation of p65 peaked at 4 hours and persisted up to 24 hours, indicating sustained NF-κB activation. In contrast, under hypoxia, nuclear translocation of NF-κB became detectable only at 24 hours, occurring later than the stabilization and nuclear accumulation of HIF-1α. These findings suggest that LPS induces early and sustained NF-κB activation that precedes Hif-1α transcriptional upregulation, whereas hypoxia triggers a delayed NF-κB response relative to HIF-1α activation.


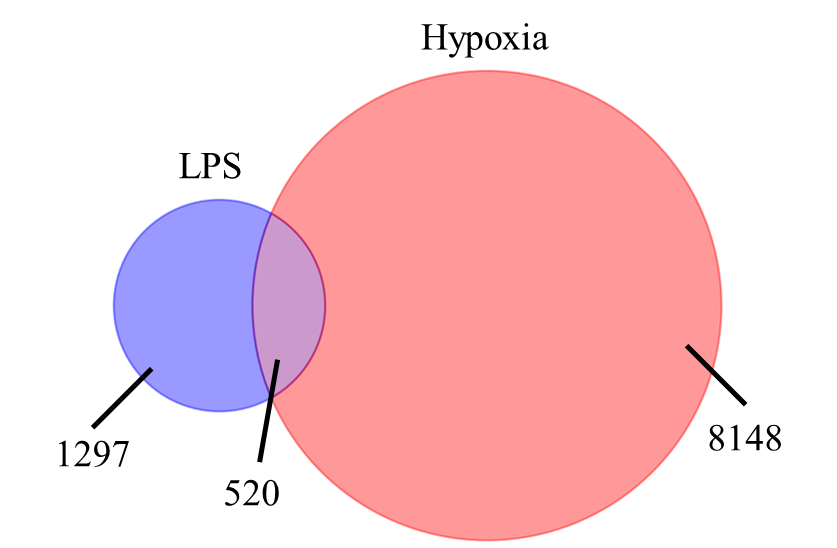
**Figure S3. Venn diagram showing overlap of HIF-1α binding peaks under LPS stimulation and hypoxic exposure.**

Chromatin immunoprecipitation sequencing (ChIP-seq) was performed using nuclear extracts from thioglycollate-elicited peritoneal macrophages (TEPMs) treated with 1 μg/mL LPS for 24 hours or cultured under 1% O₂ for 4 hours. Sequencing reads were aligned to the mouse reference genome (GRCm38) using BWA (v0.7.17), and PCR duplicates were removed with Picard tools. Reads with mapping quality < 20 were excluded using SAMtools (v1.10). Peak calling was conducted using the HOMER suite (findPeaks, false discovery rate = 0.0001) with reads from HIF-1α–deficient samples used as control. Peaks with scores > 20 were considered definitive HIF-1α–binding sites and used for quantitative analysis. LPS stimulation induced 1,297 HIF-1α peaks, whereas hypoxia induced 8,148 peaks, with 520 peaks overlapping between the two conditions. These results demonstrate that LPS and hypoxia elicit largely distinct HIF-1α binding profiles, showing limited overlap between their respective cistromes.


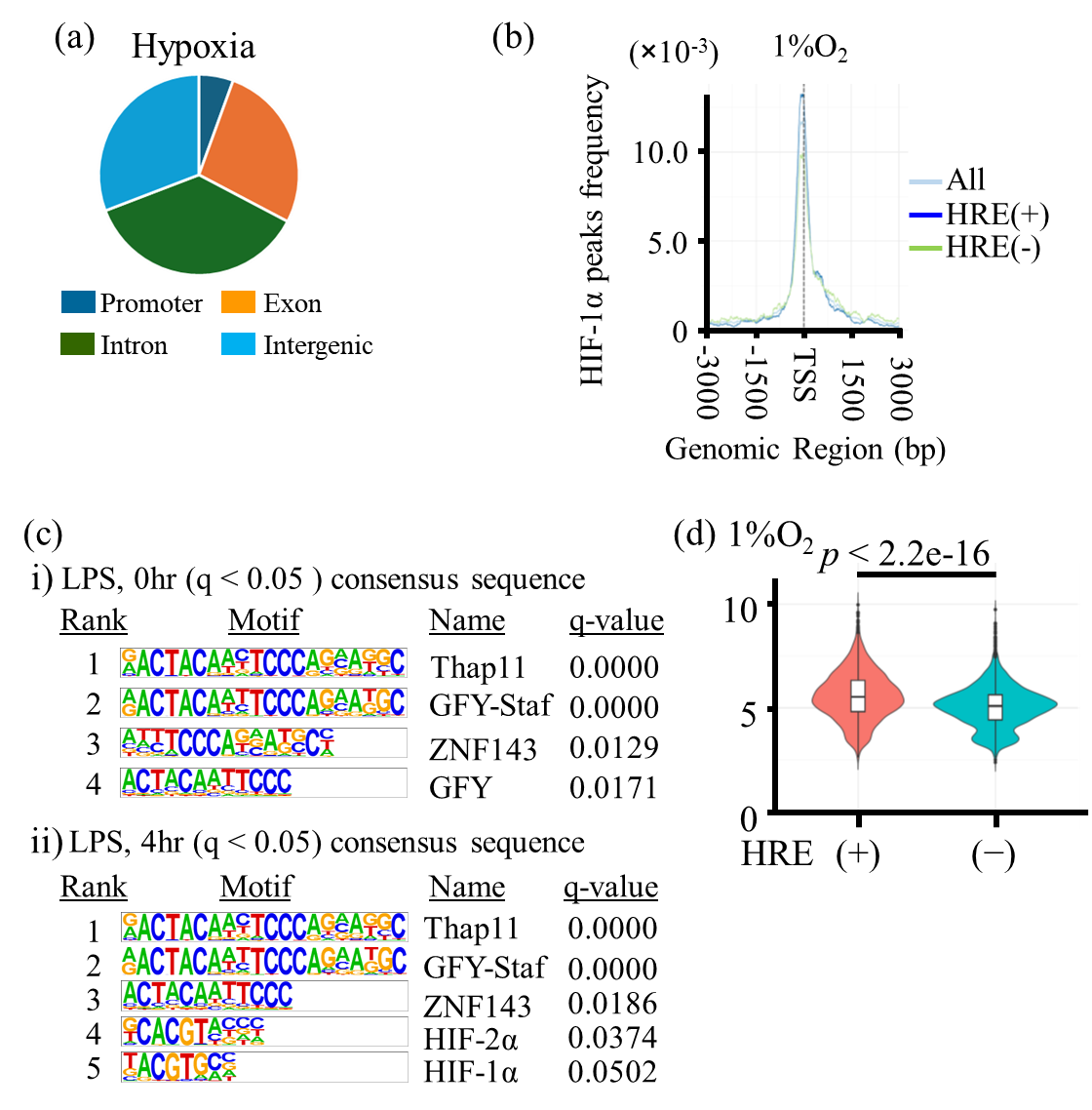
**Figure S4. Genome-wide ChIP-seq analysis of HIF-1α binding under hypoxia and LPS stimulation.**

(a) Genomic distribution of HIF-1α–binding sites identified by ChIP-seq under hypoxic conditions. Peaks were categorized according to genomic features using the HOMER annotation tool (annotatePeaks.pl) with the GENCODE vM25 annotation. Percentages of peaks within promoter (±1.5 kb from the transcription start site; TSS), exon, intron, and intergenic regions are shown. (b) Average profiles of HIF-1α binding around TSSs (−3 kb to +3 kb). Average ChIP-seq signals were calculated for all HIF-1α peaks as well as for HRE-containing (HRE(+)) and non-HRE (HRE(-)) groups under hypoxia (4 h, 1% O₂). (c) Transcription factors enriched in HIF-1α–bound motifs at 0 and 4 hours after LPS stimulation. De novo motif discovery was performed with HOMER (findMotifsGenome.pl) using the top 20 HIF-1α peaks at each time point. The top four to five enriched transcription factor motifs are displayed as sequence logos. (d) Comparison of HIF-1α peak score distributions between HRE⁺ and HRE⁻ regions under hypoxia. Violin plots show log₂-transformed ChIP-seq peak scores generated by HOMER (findPeaks, FDR = 0.0001). HIF-1α peaks were classified based on overlap with canonical hypoxia response elements (HREs; 5′-RCGTG-3′) identified using annotatePeaks.pl.


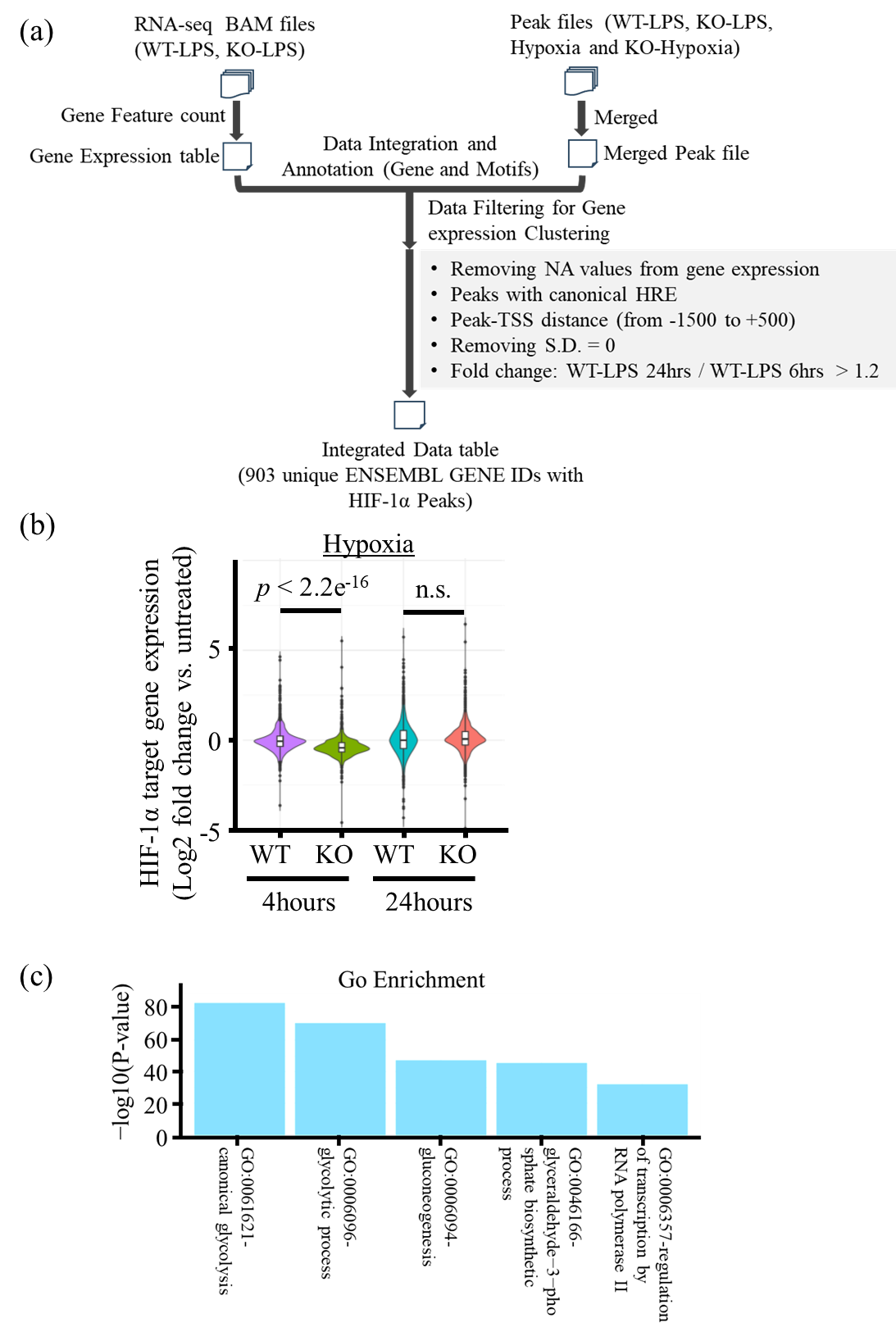
**Figure S5. Integrated analysis of hypoxia-responsive HIF-1α target genes.**

(a) Schematic overview of the integrative analysis combining ChIP-seq and transcriptomic profiling. HIF-1α peaks identified by HOMER (findPeaks, FDR = 0.0001) were linked to nearby genes, and the resulting binding data were merged with RNA-seq–derived gene-expression values (TPM, log₂-transformed) obtained using the edgeR package. (b) Comparison of hypoxia-induced HIF-1α target-gene expression between control and HIF-1α-deficient TEPMs. Violin plots show relative expression levels normalized to untreated TEPMs. Hypoxia-responsive HIF-1α target genes were defined as genes exhibiting HIF-1α binding at promoter HREs (5′-RCGTG-3′). (c) Gene Ontology (GO) enrichment analysis of Cluster 4-2 (C4-2) genes performed with the DAVID annotation tool. The five significantly enriched GO terms are shown, and bar heights represent log10-transformed adjusted p-values.


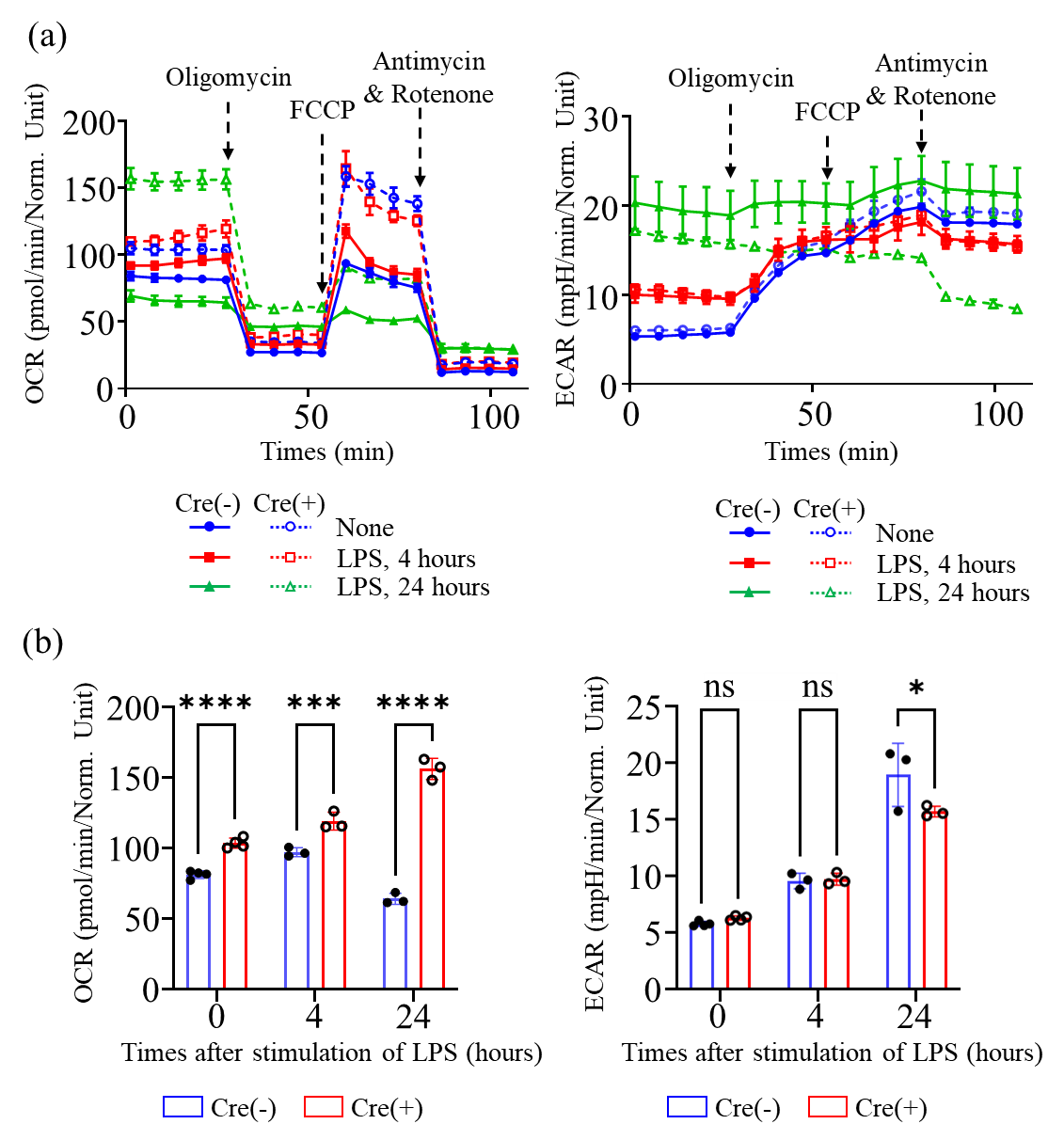
**Figure S6.** **Metabolic flux analysis of TEPMs from Tie2-HIF-1α mice after LPS stimulation.**

(a) Oxygen consumption rate (OCR, left) and extracellular acidification rate (ECAR, right) were measured in thioglycollate-elicited peritoneal macrophages (TEPMs) from control and Tie2-Cre–mediated *Hif-1α*-deficient (HIF-1α KO) mice after LPS stimulation for 0, 4, or 24 hours using Seahorse XF24 Extracellular Flux Analyzer (Agilent Technologies, USA). In control TEPMs, OCR was markedly reduced at 24 hours, whereas OCR remained elevated in HIF-1α KO TEPMs at all time points. ECAR was elevated in control TEPMs but significantly reduced in HIF-1α KO TEPMs at 24 hours. (b) Basal OCR and ECAR values are shown as bar graphs. Two-way ANOVA with Šídák’s multiple comparisons test revealed significantly higher OCR in HIF-1α KO TEPMs compared with controls at each time point, with OCR decreasing in controls but increasing in KO cells at 24 hours. For ECAR, no differences were observed at 0 and 4 hours, whereas a significant reduction was detected in HIF-1α KO TEPMs at 24 hours. Data are presented as mean ± SD. Asterisks indicate statistical significance: * *p* < 0.05, *** *p* < 0.0005, **** *p* < 0.0001.


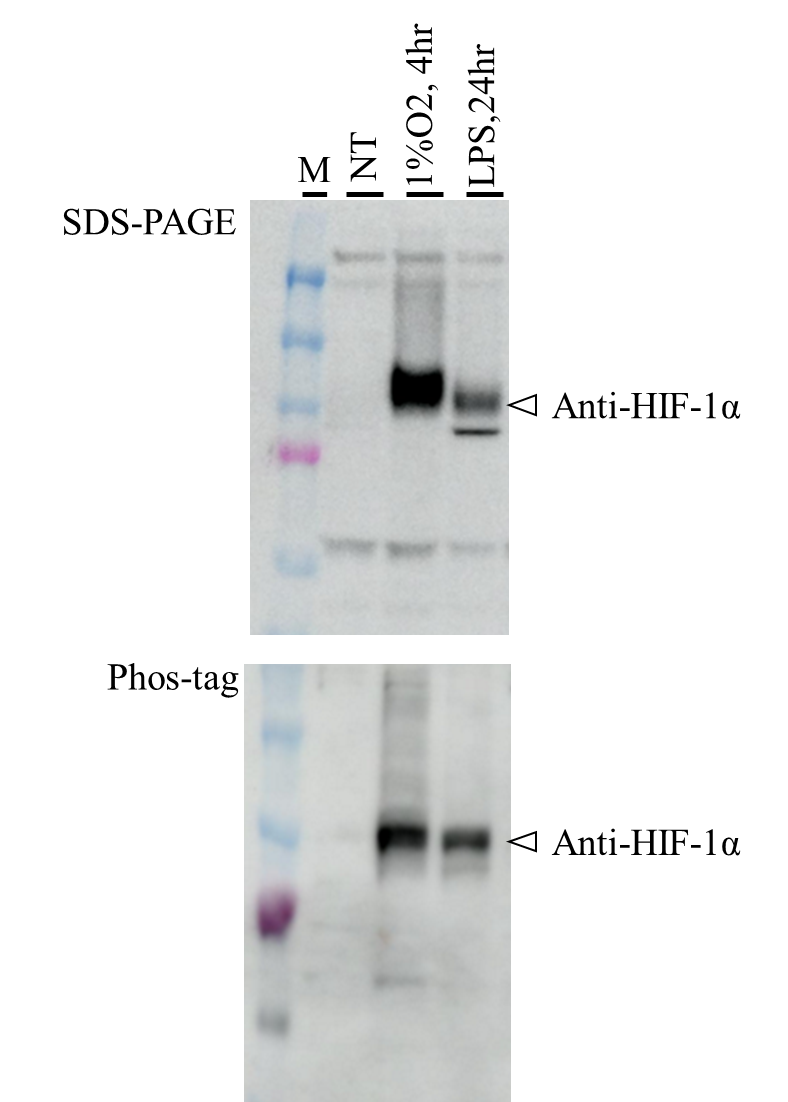
**Figure S7.** **Comparison of HIF-1α phosphorylation under hypoxia and LPS stimulation.**

(Top) Western blot analysis of HIF-1α in nuclear extracts from thioglycollate-elicited peritoneal macrophages (TEPMs) treated with vehicle (unstimulated), 1% O_2_ for 4 hours, or 1 μg/mL LPS for 24 hours. Proteins were separated by standard SDS–PAGE and probed with an anti–HIF-1α antibody. (Bottom) Phos-tag SDS–PAGE was performed using SuperSep™ Phos-tag gels (FUJIFILM Wako Pure Chemical Corporation) to detect phosphorylated HIF-1α. Both hypoxia and LPS induced HIF-1α phosphorylation; however, a distinct mobility shift was observed only under hypoxia in standard SDS–PAGE, suggesting that hypoxia promotes site-specific phosphorylation associated with a conformational change in HIF-1α.
